# ID 146 - Glossário de Avaliação de Tecnologias em Saúde (ATS) para Magistrados e Operadores do Direito

**DOI:** 10.5327/2237-9622.2025.v34s1.146

**Published:** 2025-11-25

**Authors:** Ana Luiza Cabrera Martimbianco, Carolina de Oliveira Cruz Latorraca, Isabela Porto de Toledo, Verônica Colpani, Rafael Leite Pacheco, Roberta Borges Silva, Rachel Riera

**Keywords:** judicialização da saúde, tradução do conhecimento, glossário, Avaliação de Tecnologias em Saúde

## Abstract

**Introdução:**

A tradução do conhecimento científico é um processo que envolve a intercambialidade de informações entre todas as partes envolvidas nas práticas e nas políticas de saúde. No âmbito da judicialização em saúde, termos médicos e científicos estão frequentemente presentes nos autos e nos documentos considerados pelos magistrados para apoiar a decisão em um caso específico. Sendo assim, o objetivo deste estudo é apresentar o processo de desenvolvimento e os resultados preliminares de um glossário abrangente de termos científicos presentes em demandas judiciais e notas técnicas relacionadas à saúde, fornecendo definições claras e concisas para auxiliar magistrados, operadores do direito e membros dos Núcleos de Apoio ao Judiciário (NatJus) na compreensão de conceitos utilizados nas pesquisas em saúde.

**Método:**

Este projeto foi conduzido pelo Nats/NEv do Hospital Sírio-Libanês, como entrega do projeto "Apoio técnico-científico à tomada de decisão judicial em Saúde no Brasil", desenvolvido no âmbito do Programa de Apoio ao Desenvolvimento Institucional do Sistema Único de Saúde (Proadi-SUS), triênio 2024-26. A proposta para o desenvolvimento do glossário seguiu as etapas da metodologia de Design Thinking: empatia (conhecer as necessidades e as perspectivas do público-alvo), definição da questão (identificação de lacunas na compreensão dos termos médicos e científicos nas notas técnicas e demandas judiciais), ideação (análise quanto à pertinência dos termos sugeridos), prototipagem (desenvolvimento da versão-piloto do glossário), reavaliação (avaliação da aplicabilidade prática pelos usuários), implementação e melhoria contínua. A elaboração do glossário também seguiu os itens relacionados ao desenvolvimento de ferramentas da checklist Cosmin (COnsensus-based Standards for the selection of health Measurement INstruments).

**Resultados:**

Oitenta e quatro magistrados, operadores do direito e membros dos NatJus preencheram um formulário on-line, com questões relacionadas ao formato de apresentação do glossário e à indicação livre de termos. A maioria dos respondentes (61%) optaram pela organização dos termos em ordem alfabética e por grupos temáticos; 63% preferem que os termos sejam descritos de forma sucinta, mas com a inclusão de exemplos e links para mais informações. Os termos indicados, considerados relevantes e condizentes com a proposta do glossário, foram analisados e selecionados para inclusão. Os termos mais frequentes foram: análise de impacto orçamentário, avaliação de tecnologias em saúde, avaliação econômica, custo-efetividade, custo-benefício, ensaio clínico randomizado, GRADE, intervalo de confiança de 95%, qualidade de vida ajustada por anos (QALY), real-world evidence, revisão sistemática, risco relativo, entre outros. As definições de cada termo foram estabelecidas e o protótipo do glossário foi enviado a dois magistrados e dois membros do NatJus para reavaliação. A versão final do glossário será disponibilizada em formato digital e será atualizado periodicamente.

**Conclusão:**

O glossário de termos técnico-científicos sobre ATS foi projetado para facilitar a compreensão e a interpretação de processos judiciais relacionados à saúde. Espera-se que o glossário possa preencher lacunas do conhecimento e familiarizar os magistrados, operadores do direito e membros dos NatJus com o vocabulário e os conceitos utilizados nas pesquisas científicas em saúde, contribuindo dessa forma para a tomada de decisões judiciais mais fundamentadas e justas.

